# Opposing effects of reward and punishment on human vigor

**DOI:** 10.1038/srep42287

**Published:** 2017-02-13

**Authors:** Benjamin Griffiths, Ulrik R. Beierholm

**Affiliations:** 1 Centre for Computational Neuroscience and Cognitive Robotics, University of Birmingham, Birmingham, UK; 2 Department of Psychology, Durham University, Durham, UK

## Abstract

The vigor with which humans and animals engage in a task is often a determinant of the likelihood of the task’s success. An influential theoretical model suggests that the speed and rate at which responses are made should depend on the availability of rewards and punishments. While vigor facilitates the gathering of rewards in a bountiful environment, there is an incentive to slow down when punishments are forthcoming so as to decrease the rate of punishments, in conflict with the urge to perform fast to escape punishment. Previous experiments confirmed the former, leaving the latter unanswered. We tested the influence of punishment in an experiment involving economic incentives and contrasted this with reward related behavior on the same task. We found that behavior corresponded with the theoretical model; while instantaneous threat of punishment caused subjects to increase the vigor of their response, subjects’ response times would slow as the overall rate of punishment increased. We quantitatively show that this is in direct contrast to increases in vigor in the face of increased overall reward rates. These results highlight the opposed effects of rewards and punishments and provide further evidence for their roles in the variety of types of human decisions.

Decision making in complex scenarios involves not only choosing between different options (e.g. a cat choosing what mouse to catch) but also choosing the speed of the action. Too slow a response can lead to lost opportunities (mouse escapes) while too fast a response can be metabolically demanding. Regulating the speed of a behavioral response (vigor) can be framed as a separate decision making problem, dependent on the environment and its potential rewards and punishment. An environment rich in potential rewards imposes a high opportunity cost for inaction (fewer mice caught or the mice all escape), while an environment with potential dangers (dogs around every corner) leads to a benefit of inaction. This is the basic idea behind a theoretical account of vigor^1^ that leads to quantitative predictions for the modulation of mammalian response speed (and its inverse: reaction time) by both rewards and punishments^2^,^3^.

While the model was originally derived for the case of rewarded behaviour, here we briefly extend it to punishment and apply it to a specific reaction time task (see Methods). In short, the model predicts that increased rates of rewards due to a potential opportunity cost to sloth should lead to increased vigor/reduced reaction times while high rates of punishment (due to high ‘opportunity gain’) should lead to slower performance.

Previous results^4^ have shown how human subjects indeed modify their reaction times based on the experienced rate of rewards, speeding up when the rate is high and slowing down when the rate is low. Studies in humans^5^ as well as rats^6^ have recently shown a clear link between dopamine levels and reward related vigor, further supporting the proposed influence of rewards on vigor mediated by dopamine.

High rates of punishments (through aversive stimuli or monetary loss) in contrast are known to have inhibitory effects on behavior, even leading to learned helplessness (a model of depression) if no escape is possible^7^. Theoretical accounts of this effect appeal to a decrease in opportunity cost; if actions lead to potential punishments, actions should advantageously be delayed^2^,^3^,^8^. Experiments on the threat of punishment (as opposed to punishment rate) have had mixed results, with some showing inhibitory^9^,^10^ and others excitatory effects on vigor^11^.

In order to examine this proposed role of punishment on vigor we asked human subjects to perform a speeded reaction time task in order to avoid punishment (losing money). If punishment is escapable through swift action, subjects should decrease reaction times as the threat of punishment increases. However, the deferment of future punishments may conversely lead to a slowing down as the punishment rate increases. By comparing the outcome with our previous results from the rewarding condition^4^ we aimed to gain a better understanding of the factors affecting human vigor.

## Results

**Figure 1:**
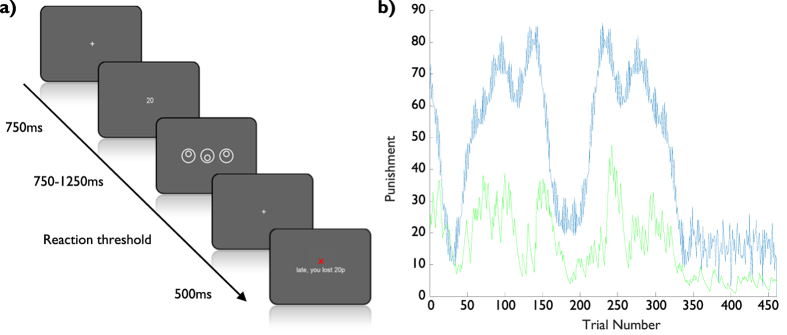
(**a**) Trial layout. After being shown the potential monetary deduction, participants had to identify the ‘odd one out’ within a personal reaction threshold. Feedback was given 500 milliseconds later. (**b**). The fluctuation of potential punishment on the current trial (blue) and averaged punishment (green, median alpha 0.13) in the form of monetary deduction across trials.

We asked human subjects to perform a speeded reaction time task, involving recognizing the oddball out of three stimuli (see Fig. 1a). Subjects had been given an initial monetary endowment of £5 and were informed that they could keep the money minus the amounts they would lose in the task. If subjects, by button press, were able to indicate the odd-ball stimulus within the time limit (set individually) they would incur no loss. If, however they pressed the wrong button or were too slow to respond they would lose an amount of money specific to the current trial. The potential punishment (available punishment, AP) was indicated at the beginning of each trial (see Fig. 1b).

**Figure 2:**
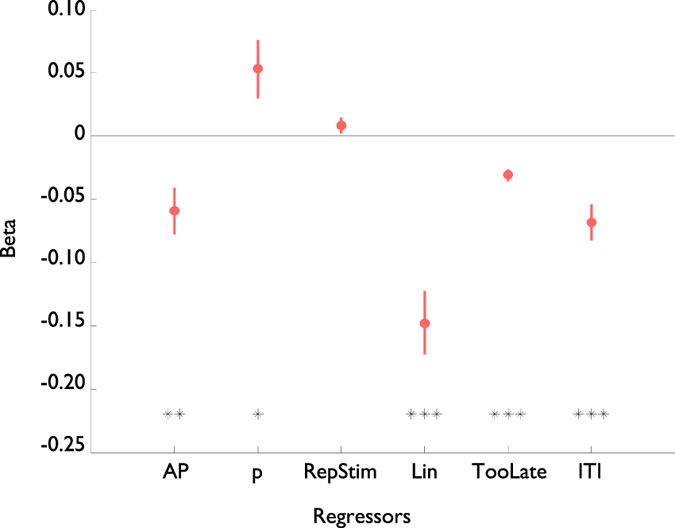
Beta values (mean and standard errors) for different regressors, based on punishing subjects for being slow or incorrect. *p < 0.05, **p < 0.01, ***p < 0.001.

We fitted a linear regression model to the log-transformed reaction times, including fitting the learning rate for the averaged punishment (***α*** = 0.314 (+ −0.398), median ***α*** = 0.130). Performing a 2-sided t-test across subjects on the beta values from the regression showed that while the effect of the Available Punishment (AP, see Fig. 2) was significantly negative, i.e. sped up response (p < 0.01, t = −3.24, dof = 21), the effect of the Averaged Punishment, (p), was significantly positive (p < 0.05, t = 2.28), implying that subjects would slow down as punishments accumulated. This was in accordance with the expectations from computational theory^1^,^3^ (see also Methods).

For other regressors, significant negative effects were found for a Linear component (p < 0.001, t = −5.95), whether the previous trial had been Too Late (p < 0.001, t = −6.69) and for larger inter-trial intervals (ITI, p < 0.001, t = −4.83), while there was no significant effect of the repetition of stimulus (RepStim. p > 0.05, t = 1.29). Summary statisitcs of the punishment data are provided in Table 1.

**Figure 3:**
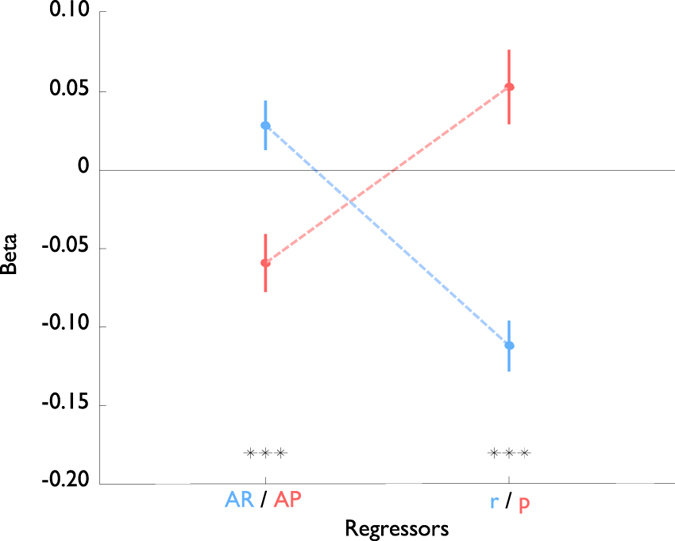
Beta values (mean and standard errors) for available reward (AR, blue) and average reward (r, blue), based on rewarding subjects for being correct and fast, plotted against available punishment (AP, red) and average punishment (p, red), based on punishing subjects for being incorrect or slow. Reward data are re-plotted from Beierholm *et al*.^5^. ***p < 0.001.

Comparing to the previous Rewarding experiment (ref. 4, reanalysed in ref. 5) allows us to examine the differential effects of rewards and punishments on vigor (Fig. 3). Using a 2-sample t-test we found no significant difference across the two experiments for the Linear (p > 0.05, t = −0.46), Intertrial intervals (p > 0.05, t = 0.95), with a slight significant difference for Too Late (p < 0.05, t = −1.95). The fitted learning rates were also not significantly different (p > 0.05, t = 1.78).

Critically, the Available Reward/Punishment and averaged Reward/Punishment both switched signs, with a significant difference (p < 0.001, t = −3.52 for AP/AR, p < 0.001, t = 5.96 for averaged Reward/Punishment), showing opposite effects of rewards and punishments on the subject vigor. The only other regressor to show such a strong difference was the Repetition of Stimulus (p < 0.001, t = 6.82), which surprisingly had no effect on behaviour in the Punishment experiment.

## Discussion

In a reaction time task, we showed that the effects of punishments on human vigor are quite distinct from those of reward: a high rate of punishment causes a decreased vigor (longer reaction times) even though the instantaneous threat of punishment increases vigor. In contrast, a high reward rate causes increased vigor (shorter reaction times) while the instantaneous potential reward caused a small decrease in vigor.

The threat of a punishment thus causes subjects to speed up their response, seemingly making it a good way to motivate fast reaction times by subjects. However, in accordance with theoretical modelling^3^ (see Methods below) the prolonged exposure to punishment leads to an overall slowdown in responses. When the response is followed by a potential punishment subjects have an implicit incentive to slow down in order to delay the punishment, leading to a potential conflict between the avoidance of the punishment and its delay. Our results thus confirm the model’s prediction. This is also in accordance with a large amount of previous work on punishment (see Gray and McNaughton^12^) which shows behavioural inhibition for responding when placed under aversive conditions.

It should be emphasized that this is a non-instrumental effect; slowing down for large levels of punishment can lead to larger levels of punishment within this task. Nevertheless the inhibitory aspect of the punishments is similar to a Pavlovian-Instrumental interaction^13^, the task-dependent Instrumental response is influenced by the non-specific behavioural inhibition due to the punishment. Our work also complements that of Dayan^8^ which extended the model^1^ to include stochastic effects and arming time of response but did not address the problem of stochastic responses themselves.

When analysing the punishment data, we found that the RepStim regressor (reflecting the repetition of stimulus) did not have a significant effect on reaction times, in contrast with the rewarding experiment. The reason for this is unclear, but may be due to the different expectations of the subjects across the two experiments, leading to less priming of responses for punishing stimuli than rewarding.

Experiments on human reaction times have often found potential trade-offs between being fast and accurate responses (see Gold and Shadlen^14^ for a review), a phenomenon that could provide an alternative explanation for the effect of available punishment (AP) that we found. According to this idea, as AP increases subjects might speed up, at the cost of lower accuracy. However, similar to our previous reward-based experiments^4^,^5^ we found no correlation between the available punishment (AP, identical for all subjects) and the average proportion of correct responses performed across subjects (r = −0.021, p > 0.05) implying that subjects were not modulating their responses in order to trade-off speed and accuracy. Likewise comparing AP for erroneous trials against correct trails within all subjects revealed no difference (2-sample t-test, t = 0.532, p > 0.05). A subject aware of their individual risk of performing errors as a function of reaction time could of course incorporate this knowledge into their decision making, a potential topic for future study.

A further potential caveat could be that the average punishment rate could be driven by trials where subjects make a button error, leading subjects to naturally slow down. If this were the case we would expect to find that subjects with more errors (or more errors relative to number of too late responses) would show a larger effect of the average punishment rate. We therefore correlated across subjects the number of errors, as well as the ratio of number of errors to the number of trials with too late a response, with the beta value for the averaged punishment but found no significant effect (respectively r = 0.179, p > 0.05 and r = 0.143, p > 0.05). See the Supplementary Online Material for further discussion of this point.

One puzzling result is the slight decrease in vigor (longer reaction times) for the available reward (AR). While this may be related to the well documented ‘Undermining’ effect on intrinsic motivation due to external rewards^15^,^16^, this result seems counter intuitive. Not only are monetary incentives the main motivator in many types of employment, but previous tasks have indeed found reaction time decreases for monetary reward conditions^17^,^18^. There are differences between our tasks in terms of structure, reward sizes and the use of blocked trials, and we are currently investigating this through a modified task that more closely resembles these earlier studies.

While we have previously shown a link between the average reward rate and dopamine^5^, corroborating the theoretical model which predicts that tonic dopamine should encode the average reward rate, the implications for punishment are less clear. Theoretical work has suggested a link between average punishment rate and serotonin^2^,^3^, based on serotonin’s role in reduction of impulsivity. According to this idea, phasic serotonin encodes an aversive prediction error while tonic serotonin encodes the temporal benefit of sloth, i.e. the potential decrease in aversive punishments due to lack of activity (inverse to the role of dopamine).

At the same time serotonin has also been associated with depression (often clinically treated with selective serotonin reuptake inhibitors) through a number of studies^19^,^20^,^21^. Recent optogenetic results^22^ have found activation in serotonergic neurons for both rewards and punishment, thus the effects of serotonin are more elusive. Future work will look into the proposed link between the averaged punishment rate, serotonin and reduced vigor.

While we have here framed our results in terms of a specific theoretical account, the predictions are not unique as other subsequent models^23^ could potentially explain the observations equally well.

Overall, we find opposing effects of the threat of immediate punishment and rate of punishment on human vigor, a result that is a reversal of what was previously found for rewards but is in accordance with theoretical models. The implication here is that while the threat of punishment may cause subjects to speed up on a reaction time task, the long-term effects are deleterious and can lead to overall decreases in speed. Future work will examine what implications this may have for vigor and motivation in tasks that are not specifically designed to encourage fast reaction times but for which speed is nevertheless important.

## Methods

### Punishment Experiment

#### Participants

22 participants were recruited from the University of Birmingham psychology participation pool composed primarily of psychology under-graduate students. In addition to a performance-dependent financial payment, participants received course credit for participation. Due to flashing on-screen images, individuals with epilepsy or prone to migraines were excluded from the experiment. Prior to commencing the experiment, participants received clear instructions and provided written informed consent. Ethical approval was granted by the University of Birmingham Research Ethics Committee, complying with the Declaration of Helsinki.

#### Procedure

Participants completed an oddball discrimination task similar to that of Guitart-Masip, Beierholm, Dolan, Duzel and Dayan (2011). The experiment was conducted using a regular PC monitor and keyboard and was presented using Matlab (Mathworks, Natick, MA) and the Cogent 2000 Toolbox (Wellcome Department of Imaging Neuroscience, Institute of Neurology, London, UK). Prior to starting the main task, participants completed a short practice session in which they performed a small number of trials without financial result. The practice session familiarized the participant with the task and determined a personal reaction threshold by fitting a cumulative distribution to the responses and setting the threshold at the 50 percent correct level. This ensured sufficient losses for the main task. At the beginning of the main experiment, participants were informed that they had the opportunity to take away £5 from the session; however, incorrect or late responses during the task would lead to deductions from this sum. At the beginning of each trial (see Fig. 1a for trial layout), the participant was presented with a number representing the amount of money which could be lost during that trial. After a variable period between 750 and 1250 ms, three images were displayed on screen for which the participant had to identify the ‘odd one out’ using the corresponding button on the keyboard. If the participant chose incorrectly or failed to respond within the personal reaction threshold, a red cross appeared on screen 500 milliseconds later with the monetary sum lost as a result. The amount of the potential deduction followed a fixed function of time designed to allow the fluctuation of punishment to be independent of other influential variables (see Fig. 1b for punishment function). If participants correctly responded within the reaction threshold, a green tick was displayed 500 ms later. After a 1000 ms delay, the next trial would begin. Participants performed this task for 27 minutes, allowing a variable amount of trials to be completed (see Table 1). After finishing the task, 10% of trials were randomly selected and the money lost on these trials was subtracted from the £5 sum.

#### Analysis

Data was initially log-normalized, by assuming a distribution according to *RT~N*(log(*RT* − *c),* log(*μ), σ*^**2**^), where parameters {*c, μ, σ*} were found through maximum likelihood estimate for each subject. Note that parameter *c* encapsulates any lag unrelated to reaction time itself, e.g. perceptual processing. Further analysis were done using the log-transformed variable, log*RT* = log(*RT* − *c*).

Model based analysis was identical to previous work^4^,^5^, consisting of performing a linear regression using a set of regressors (see below), with a Bayesian prior applied to the parameter fit through Expectation Maximisation. In short this allowed the average group parameters to function as regularisers on the fit of individual subject parameters, thus avoiding overfitting.

Regressors, *X*, were:

- Available punishment: The amount the subject might lose in a given trial (AP), displayed at beginning of the trial.

- Averaged punishment: An exponentially discounted average over previous actual punishments incurred (p), calculated as: *p(i)* = *p(i* − *1)* + α*(Pun(i)-p(i)), where *Pun(i)* is the punishment incurred in trial *i* and the discount rate (learning rate) *α* was fit individually to the behaviour of each subject (see below).

- Repetition of stimulus: A binary indicator {0, 1} specifying if the stimulus in trial *i* was identical to the stimulus in trial *i* − *1*.

- Linear effect: A linear term.

- Too late: A binary indicator {0, 1} specifying if subjects had been too late in trial *i* − *1* (which may cause them to speed up in trial *i*).

- Inter trial interval: The amount of time between the presentation of the potential punishment and the presentation of the stimulus (750–1250 ms).

In addition, a constant term was used to account for the average of the response times. All regressors were normalized with mean zero and variance one. While the Available Punishment and Averaged Punishment were our natural regressors of interest, the remaining regressors were included as nuisance variables that might be able to explain aspects of the variance of the model.

As per standard linear regression we assumed a linear model for the log-transformed reaction times log*RT* ~ X*β + ε, where **ε** is normal distributed random noise. After finding the maximum likelihood of the β parameters we used a Laplace approximation around this parameter set to estimate the variance around the fit (see Beierholm *et al*.^5^ for details of this procedure). This allowed us to approximate a normal distributed likelihood function for the parameters *P*(log*RT|β*). Given a prior *P*(β), we can find a posterior estimate of the parameters 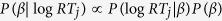 for each subject j. This prior *P(β)* can be estimated iteratively (by Expectation Maximization) by repeatedly setting 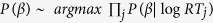, (ie maximizing the likelihood of the prior across subject), and recalculating *P(β|*log*RT*_*j*_) until convergence^5^,^24^. While the prior allows the individual subject parameter fits to be regularized (in a Bayesian sense), the prior itself is estimated by maximum likelihood across subjects.

#### Reward experiment

The methods for the rewarding experiment as well as the analysis of the data have been described elsewhere^5^. For ease of reading we summarize it here. In short, 39 subjects were recruited and given an oddball task (as above) with the potential of winning money. The experiment was identical to the punishment condition but with a correct response within the time limit leading to the potential rewarding of money, as opposed to the potential deduction of money. The potential winnable trial reward was identical to the amount of money that could be lost in the punishment condition. Display, timing etc. were otherwise identical. Note that using a within-subject design for rewards and punishment was not possible due to potential learning effects; being exposed to the reward/punishment function would influence subject behaviour in the later secondary experiment.

Analysis was identical to the punishment condition with regressors: ‘Available reward’, ‘Averaged reward’, ‘Repetition of stimulus’, ‘Linear effect’, ‘Too late,’ ‘Inter trial interval’, as well as a constant term.

#### Model derivation for Punishment task

**Figure 4:**
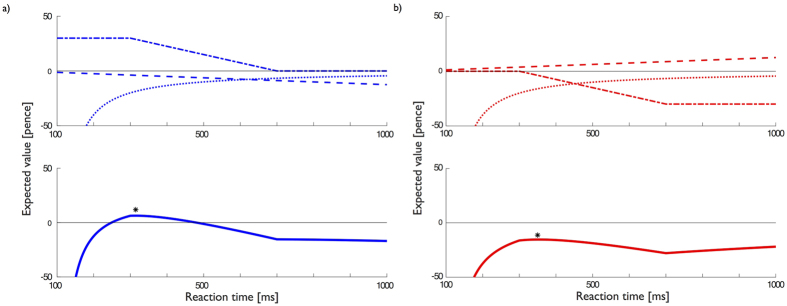
(**a**) Punishment (red) and (**b**) Rewards (blue) influence the optimal response times for subjects. Top: An example of how the available punishment (reward), opportunity gain (cost) and movement cost add together to produce the total Expected value (Bottom) of making a reaction at time *τ*. Optimal behavior entails maximizing this function, indicated by *In each figure. For this figure parameters were K = 4000 ms*pence, a = 200 ms, t = 500 ms, rho = 0 pence. We assumed 100 ms for perceptual processing.

We assume that subjects wish to maximize their potential payout (minimize punishment) over an unknown number of trials N. In a given trial *i* the subject can (implicitly) maximize the expected future value *V*_*i*_ of being in it’s current state *S*. Relying on results from Reinforcement Learning^25^ we can write down the Bellmann equation


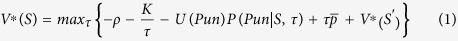


where 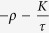 is the cost of movement consisting of a fixed and a time dependent term, *U(Pun)* is the utility of getting punishment *Pun* with probability *P(Pun|S, τ*) dependent on the state and movement time, the opportunity gain 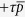 depending on the movement time and value of time (average punishments)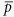. Finally 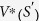 is the reward/punishment values expected at the subsequent state *S*′. We here adopt the convention of using positive values for all variables, i.e. even though punishment could be seen as a negative reward for clarity we treat it as a positive punishment. We will assume that the probability of reward does not depend on the state but that a subject has to react within a certain time limit, *t*, in order to avoid punishment. However the motor system adds extra variability so that the motor response happens at time *τ* ± *a* (uniformly), hence the probability of punishment 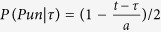 within the range *τ* ± *a* (note that the shape of this distribution is chosen primarily for mathematical convenience). Note that larger temporal motor noise requires the subject to shift responses earlier for the same level of performance and leading to a lower average latency. An example of how the different contributions add together to produce the expected reward as a function of the reaction time is given in Fig. 4.

Maximising the expected value (minimizing expected punishment) with regard to planned response time *τ* is done through finding the derivative:


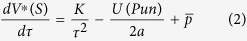


Setting this equal to zero and solving for *τ* gives the solution


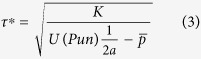


In other words, the optimal response time should decrease as the threat of punishment, *U(Pun)*, goes up, but increase with the average rate of punishment 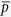. Note that the specific shape of this function (e.g. the square root function) depends on the details of our initial model assumptions.

#### Model derivation for Reward task

For completeness we include the derivation of the model for the reward task. We again assume that subjects wish to maximize their potential payout (maximize reward), leading to the Bellmann equation


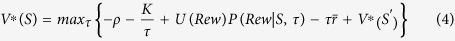


where in analogy with the model above 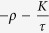 is the cost of movement consisting of a fixed and a time dependent term, *U(Rew)* is the utility of getting reward *Rew* with probability *P(Rew|S, τ*) dependent on the state and movement time, and the opportunity cost 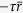 depending on the movement time and value of time (average reward rate)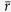. With the same assumptions as above (state independence, uniform temporal motor noise) the probability of reward is 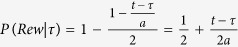 within the range *t* ± *a*.

Maximising the expected value is again done by finding the derivative:


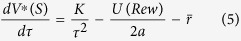


Setting this equal to zero and solving for *τ* gives the optimal solution


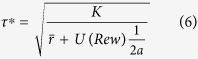


In other words, just as for punishment the response times should decrease as the potential reward, *U(Rew)*, goes up, but contrary to the punishment case the reaction times should decrease with the average rate of reward 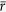.

## Additional Information

**How to cite this article:** Griffiths, B. and Beierholm, U. R. Opposing effects of reward and punishment on human vigor. *Sci. Rep.* **7**, 42287; doi: 10.1038/srep42287 (2017).

**Publisher's note:** Springer Nature remains neutral with regard to jurisdictional claims in published maps and institutional affiliations.

## Supplementary Material

Supplementary Online Material

## Figures and Tables

**Table 1:** 

Mean number of trials performed	Mean number of Correct and within time limit (not punished)	Mean number of Wrong (punished)	Mean number of Late (punished)	Mean response time [ms]
458.6 (+−2.3)	239.5 (+−41.2)	44.9 (+−20.1)	174.1 (+−34.2)	399.8 (+−30.4)

Summary statistics for Punishment data, mean (+− standard deviation).
