# Supplementary material for: Opposing effects of reward and punishment on human vigor
Source: Sci Rep. 2017 Feb 13;7:42287. doi: 10.1038/srep42287 (PMC5304224; doi:10.1038/srep42287)
Supplement: Supplementary Online Material [file srep42287-s1.doc]

**Opposing effects of reward and punishment on human vigor**

**Supplementary material**

**Benjamin Griffiths^1^ & Ulrik R. Beierholm^1,2^,***

1. Centre for Computational Neuroscience and Cognitive Robotics,

University of Birmingham, Birmingham, UK

2. Department of Psychology, Durham University, Durham, UK

*Corresponding author: [ulrik.beierholm@durham.ac.uk](mailto:ulrik.beierholm@durham.ac.uk)

## Effect of speed-accuracy trade-off on reward rate

1) In order to further examine the role of errors on punishment and reaction times we performed an extra control analysis. If the speed-accuracy strongly influences the average punishment rate then it could be possible that any effect of punishment rate is merely due to the speed-accuracy tradeoff. We removed the effect of punishments due to errors from the calculation of the of the punishment rate (counterfactual to the experimental procedure but as a control) and redid the Expectation Maximization analysis for the punishment data. While there was slight quantitative changes, the overall results did not change (see figure below), including statistical significance (2-sided t-test).


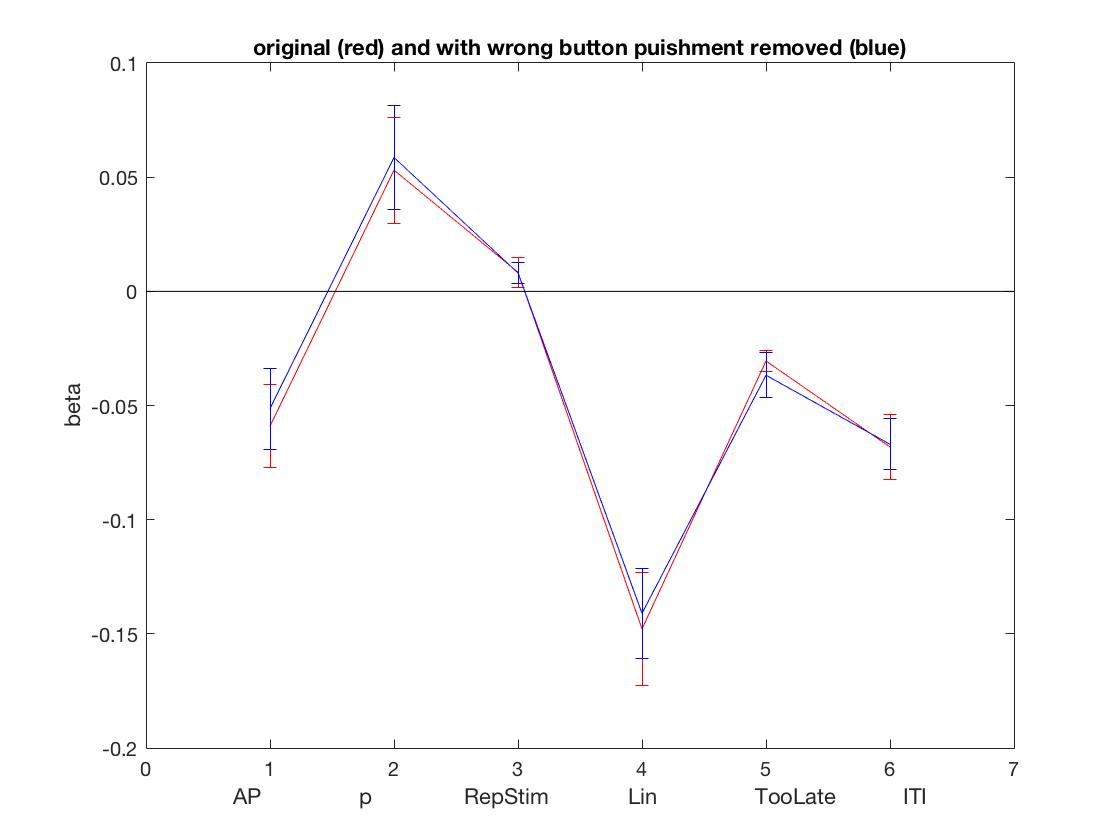


| Regressor | AP | p | RepStim | Lin | TooLate | ITI |
| --- | --- | --- | --- | --- | --- | --- |
| t (p) | -2.8923 (p<0.01) | 2.5876 (p<0.05) | 1.7128 (p>0.05) | -7.1024 (p<0.001) | -3.7174 (p<0.01) | --6.0203 (p<0.001) |

2) Likewise for the reward data, if reward rate is driven by the difference between correct and erroneous response then the causal links should be cut if the rewards were the same for correct and erroneous responses. Redoing the Expectation Maximization regression with rewards given for wrong button presses (counterfactual to the experiment but as a control) we found, as above, a small quantitative change, but similar results in terms of the Available Reward and averaged Reward regressors (see figure below).


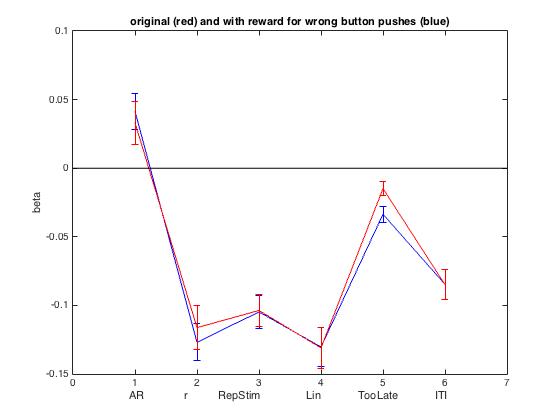


| Regressor | AR | r | RepStim | Lin | TooLate | ITI |
| --- | --- | --- | --- | --- | --- | --- |
| t | 3.1494 (p<0.01) | -9.4088 (p<0.001) | -8.7975 (p<0.001) | -9.1467 (p<0.001) | -5.7012 (p<0.001) | -7.8039 (p<0.001) |
